# ATP Hydrolysis Determines Cold Tolerance by Regulating Available Energy for Glutathione Synthesis in Rice Seedling Plants

**DOI:** 10.1186/s12284-020-00383-7

**Published:** 2020-04-09

**Authors:** Pinghui Yu, Ning Jiang, Weimeng Fu, Guangjie Zheng, Guangyan Li, Baohua Feng, Tingting Chen, Jiaying Ma, Hubo Li, Longxing Tao, Guanfu Fu

**Affiliations:** National Key Laboratory of Rice Biology, China National Rice Research Institute, Hangzhou, 310006 China

**Keywords:** ATP hydrolysis, Antioxidation, Cold stress, Energy status, Glutathione, *Oryza sativa*

## Abstract

**Background:**

Glutathione (GSH) is important for plants to resist abiotic stress, and a large amount of energy is required in the process. However, it is not clear how the energy status affects the accumulation of GSH in plants under cold stress.

**Results:**

Two rice pure lines, Zhongzao39 (ZZ39) and its recombinant inbred line 82 (RIL82) were subjected to cold stress for 48 h. Under cold stress, RIL82 suffered more damages than ZZ39 plants, in which higher increases in APX activity and GSH content were showed in the latter than the former compared with their respective controls. This indicated that GSH was mainly responsible for the different cold tolerance between these two rice plants. Interestingly, under cold stress, greater increases in contents of carbohydrate, NAD(H), NADP(H) and ATP as well as the expression levels of *GSH1* and *GSH2* were showed in RIL82 than ZZ39 plants. In contrast, ATPase content in RIL82 plants was adversely inhibited by cold stress while it increased significantly in ZZ39 plants. This indicated that cold stress reduced the accumulation of GSH in RIL82 plants mainly due to the inhibition on ATP hydrolysis rather than energy deficit.

**Conclusion:**

We inferred that the energy status determined by ATP hydrolysis involved in regulating the cold tolerance of plants by controlling GSH synthesis.

## Background

Rice is one of the most important crops in the world (Seck et al. 2012; Chauhan et al. 2017). It plays an important role in Asian grain production, which has made a great contribution to food security (Aryal and Kandel 2017; Chauhan et al. 2017; Rahaman and Shehab 2019). As a typical subtropical or tropical crop, rice plants are always damaged by cold sress (Zhao et al. 2015; Wu et al. 2016; Chen et al. 2017). This stress is not only detrimental to plant growth and development (Su et al. 2010; Thomashow 2010; Kim et al. 2017), but also limits spatial distribution and grain productivity (Su et al. 2010; Wang et al. 2016; Uphoff and Thakur 2019). It has been reported that cold stress occurring at reproductive phase can result in a large reduction in grain yield (Thakur et al. 2010; Ghadirnezhad and Fallah 2014). Additionally, slow seedling development, yellowing, withering, reduced tillering and stunted growth are always observed under cold stress at the seedling stage (Su et al. 2010; Bonnecarrère et al. 2011; Han et al. 2017; Cong Dien and Yamakawa 2019). As a result, the cultivated area of early rice and double-season late rice is significantly reduced in China (Sun and Huang 2011; Yu et al. 2012; Wang et al. 2019).

Cold stress can directly inhibit the metabolic reaction of plants and induce osmotic, oxidative and other stresses, thus preventing the expression of all genetic potential (Chinnusamy et al. 2007; Kazemi-Shahandashti and Maali-Amiri 2018; Shi et al. 2018). Indeed, the degree of damage to rice plants usually depends on time of occurrence (growth phase), the severity of chilling, and the duration of the cold stress (Li et al. 1981; Ye et al. 2009). It has been reported that the chilling threshold temperature in the early stages of development (germination and vegetative growth) is low (10–13 °C), while during the reproductive stage, the threshold temperature of rice is high (18–20 °C) (Yoshida 1981; Ghadirnezhad and Fallah 2014). During this process, cold stress causes multiple dysfunctions at the cellular level, including membrane damage, ROS production, protein denaturation and accumulation of toxic products (Bowers 1994). As a result, many tropical or subtropical crops are damaged or killed and exhibit the symptoms of chlorosis, necrosis, or growth retardation (McDonald 2002; Sanghera et al. 2011). It is worth noting that cold-tolerant species can grow at very low temperature (Sanghera et al. 2011; Xu and Cai 2014; Liu et al. 2019). Cold acclimation has been reported to be associated with a variety of mechanisms such as gene expression, change of membrane composition, accumulation of cryoprotectants and elevation of phytohormones (Lang et al. 1994; Yadav 2010; Chen et al. 2019). Among these changes, the enhancement of antioxidant capacity, especially the glutathione (GSH), is one of the most important factors for rice plants to resist cold stress since the plants cannot survive without glutathione (γ-glutamylcysteinylglycine) or γ-glutamylcysteine containing homologues (Tausz et al. 2004; Noctor et al. 2012; Hausladen and Alscher 2017; Banerjee and Roychoudhury 2019).

The GSH is the most abundant form of organic sulphur in plants apart from that incorporated into proteins, and it predominantly presents in its reduced form (GSH), with only a small proportion present in its fully oxidised state (GSSG) (Rao and Reddy 2008; Noctor et al. 2012). Indeed, GSH is an essential metabolite with a variety of functions found in plants, such as the biosynthetic pathways, detoxification, antioxidant biochemistry and redox homeostasis (Rao and Reddy 2008; Noctor et al. 2012). The most fundamental and earliest function of glutathione is in the thiol-disulphide interaction, in which reduced glutathione (GSH) is continuously oxidized to the disulphide bond form (GSSG) that is recycled to GSH by NADPH-dependent glutathione reductase (GR) (Belorgey et al. 2013; Csiszár et al. 2016). Importantly, the function of GSH in plant development cannot be achieved by other thiols or antioxidants (Noctor et al. 2012). As an important component of the ascorbate-glutathione (AsA-GSH) cycle which consists of two dominating nonenzymatic antioxidants, GSH andAsA, and four enzymes [ascorbate peroxidase (APX), monodehydroascorbate reductase (MDHAR), dehydroascorbate reductase (DHAR) and GR], GSH is involved in the removal of hydrogen peroxide caused by cold stress (O’Kane et al. 1996; Noctor and Foyer 1998; Noctor et al. 1998). In the process of hardening, the accumulation of GSH and the ratio of glutathione reduced/oxidized as parts of a complex regulatory function enhance the frost resistance of wheat (Kocsy et al. 2000; Galiba et al. 2001; Hausladen and Alscher 2017). Similarly, the enhancement of ratios of reduced and oxidized forms of AsA (AsA/DHA) and GSH (GSH/GSSG), and the fluctuation of activities of APX, GR, and DHAR in melon seedlings are induced by melatonin under cold stress (Zhang et al. 2017). Additionally, GSH and AsA were reported to be interacted with H_2_O_2_ signaling to enhance the antioxidant capacity of tomato under cold stress (Liu et al. 2018). Indeed, GSH is also resistant to cold stress through other pathways, including redox signaling, secondary metabolism and xenobiotic detoxification (Noctor et al. 2012). These processes are high-energy cost, in which the ATP, NAD(H) and NADP(H) are involved. However, few studies have investigated the relationship between GSH metabolism and energy status in plants under cold stress, and the underlying mechanism remains unclear.

Many studies have shown that γ-glutamylcysteine synthetase (γ-ECS) and glutathione synthetase (GSH-S) play important roles in GSH synthesis in plants (Cairns et al. 2006; Pasternak et al. 2008). However, both pathways are ATP-dependent (Rennenberg 1980; Meister 1988; Mullineaux and Rausch 2005) that the synthesis of GSH is affected by the energy status in plants. Moreover, the accumulation of GSH can be regulated by GR, which can resist oxidative stress caused by cold, drought, and high light and salinity (Rao and Reddy 2008). It is worth noting that GR used NADPH as an electron donor to reduce GSSG to GSH (Rao and Reddy 2008; Noctor et al. 2012). In fact, GR utilizes NADH to catalyze the reduction of GSSG with low efficiency (Halliwell and Foyer 1978). These results suggest that the role of GSH in plants against cold stress is a high energy-consuming process. However, energy shortage is always found in plants under abiotic stress, which may impair the antioxidant capacity, especially for low resistant plants (Baena-González and Sheen 2008; Jin et al. 2015; Dahal and Vanlerberghe 2017; Asami et al. 2018; Islam et al. 2019). Therefore, energy homeostasis is critical for plants to survive in abiotic stresses, including cold stress, by accumulating GSH. In our study, two rice pure lines with different cold tolerance were selected to investigate how energy status affects the accumulation of GSH in plants, in which the antioxidant capacity, carbohydrate metabolism, GSH and energy, heat shock proteins, relative electrical conductance (REC), maximum fluorescence quantum efficiency (Fv/Fm) and actual fluorescence quantum efficiency (Y (II)) of leaves were determined.

## Methods

### Plant Materials and Growth Conditions

This study was conducted at an experimental farm at the National Rice Research Institute in Hangzhou, Zhejiang Province, China. Two rice pure lines were selected, namely Zhongzao39 (ZZ39, cold tolerant) and its recombinant inbred line 82 (RIL82, cold susceptible), which is a F9 line that was selected from the RIL population derived from the rice cross ZZ39 × ZJZ17 (Zhongjiazao17). The rice seeds were soaked for 48 h, germinated at 37 °C for 24 h, and then were sown directly in the pots (10 cm height and 10 cm diameter) in a plant growth chamber where the air temperature was controlled at 28 / 22 °C (day/night), the relative humidity was 70% under the natural sunlight of 1000 μmol m^− 2^ s^− 1^. At the six-leaf stage, two rice pure lines were subjected to cold stress for 48 h, where the stress temperature were set to be 13 / 10 °C (day/night), while the control temperature was 25 / 20 °C (day/night). During the period, the relative humidity was maintained at 70% and the light intensity was 300 μmol m^− 2^ s^− 1^. At the end of cold stress, the first fully expanded leaves were selected to determine the Fv/Fm and Y (II), and then collected to determine the REC, MDA, H_2_O_2_, chlorophyll, carbohydrates, energy metabolism and allocation, antioxidant capacity, GSH, GSH-S, GR and heat shock proteins (HSPs).

### Effect of Glutathione (GSH) and 3-Ab Alone or Together on Rice Plants under Cold Stress

In order to investigate the underlying mechanism of GSH and 3-ab functioned in cold tolerance of rice, 1 mmol·L^− 1^ GSH and 25 mmol·L^− 1^ buthionine sulfoximine (BSO, a GSH synthetic inhibitor, Noctor et al. 2012) as well as a 1 mmol·L^− 1^ Poly (ADP-ribose) polymerase (PARP) synthetic inhibitor 3-aminobenzamide (3-ab, Keppler et al. 2018) containing 0.1% (v/v) Tween20 as a surfactant were sprayed onto rice leaves with 10 mL per pot about 30 min before cold stress conducted. The first fully expanded leaf samples were collected to determine REC and MDA 48 h later. According to the above results, the synergistic effects of GSH and 3-ab on cold tolerance of rice plant were also investigated. About 30 min before the cold stress, these two chemicals containing 0.1% (v/v) Tween20 as a surfactant were sprayed on rice leaves with 10 mL per pot together. 48 h later, the first fully expanded leaves were collected to determine the H_2_O_2_, MDA, GSH and ATP levels and the contents of PARP and ATPase.

### Measurements of Chlorophyll Content and Fluorescence Quantum Efficiency

The chlorophyll concentration was measured using an ethonal extraction procedure (Sartory and Grobbelaar 1984), in which 0.1 g leaf sample was sliced and immersed in 20 mL 95% ethanol for 48 h in the dark. Chlorophyll concentration was determined at 665 nm and 649 nm using a spectrophotometer (Lambda25; Perkin Elmer, Freemont, CA, USA).

After a 30-min dark adaptation period, Fv/Fm and Y (II) of the leaves were measured using a portable chlorophyll fluorescence spectrometer (PAM-2500 chlorophyll fluorescence system; Heinz Walz, Effeltrich, Germany) (Zhang et al. 2018a).

### Relative Electrical Conductance Measurement

Following the method of Xiong et al. (2012), 0.5 g of fresh leaves were collected at the end of the cold stress, cut into 25-mm^2^ pieces, and immediately immersed into a test tube with 12 mL deionized water for 2 h at 25 °C. After incubation, a conductivity meter (DDA-11A; Shanghai Hongyi Instrument Co. Ltd., Shanghai, China) was used to measure the electrical conductivity of the solution (EC1). The electrical conductivity (EC2) was measured again after the samples were heated at 80 °C for 2 h in their effusates and cooled to 25 °C. The relative ion leakage was calculated as the ratio between EC1 and EC2.

### H_2_O_2_ Measurement

Following the method of Brennan and Frenkel (1977) with some modifications, about 0.2 g of frozen leaves were homogenized in 4 mL extraction solution containing 10 mM 3-amino-1, 2, 4-triazole. The solution was centrifuged at 6000×*g* for 25 min, and then 1 mL of 0.1% titanium tetrachloride dissolved in 20% H_2_SO_4_ was added to 2 mL of the supernatant. After the reaction, undissolved material was removed from the solution, the absorbance was determined at 410 nm by a spectrophotometer (Lambda 25; Perkin Elmer, Freemont, CA, USA).

### Lipid Peroxidation Measurement

About 0.2 g of frozen leaves were homogenized in 2 mL of 5% trichloroacetic acid, and then the MDA content was estimated through determining the concentration of thiobarbituric acid reactive substances (Dhindsa et al. 1981).

### Antioxidant Enzyme Activity Measurements

The superoxide dismutase (SOD) activity was determined by the method of Giannopolitis and Ries (1977). The peroxidase (POD) activity was measured by the method of Maehly and Chance (1954), in which the guaiacol was converted to tetra guaiacol, and was monitored at 470 nm. The catalase activity (CAT) was determined using the modified method of Aebi (1974) as described by Zhang et al. (2016). The ascorbate peroxidase (APX) activity was determined by the method of Bonnecarrère et al. (2011).

### Measurements of GSH and GSSG Content and GR Activity

The glutathione content was assayed by monitoring the change in absorbance of 2-nitro-5-thiobenzoic acid at 412 nm for 5 min (Noctor et al. 2016). The GSSG content was determined by the kit from the Comin Biotechnology Co., Ltd., Suzhou, China. The GR activity was assayed by monitoring the decrease in NADPH at 340 nm for 3 min using a kit from the Beijing Solarbio Science & Technology Co., Ltd., Beijing, China.

### Carbohydrate Measurements

According to the modified anthrone-sulphuric acid colorimetric method (Dubois et al. 1956), fresh leaf samples (0.2 g) saturated in 10 ml of deionized water were boiled for 20 min to determine the soluble sugar and starch contents. To determine soluble sugar, the extract was filtered and treated with anthrone and 98% sulphuric acid, and the mixture was incubated in boiling water for 15 min. A spectrophotometer (Lambda25; Perkin Elmer, Freemont, CA, USA) was used to determine the absorbance at 485 nm. As to starch content, the sediment of the filtered extract containing sugar content was dried, weighed, and boiled with deionized water and perchloric acid at 9.2 M and 4.6 M, respectively. The supernatant was also determined by a spectrophotometer at 485 nm. The total non-structural carbohydrate (NSC) was calculated as the sum of soluble sugar and starch contents.

### NAD(H) and NADP(H) Measurements

The NAD(H) and NADP(H) were extracted with 1 mL 0.1 M HCl or 0.1 M NaOH, respectively (Matsumura and Miyachi 1980). For NAD(H) including NAD^+^ and NADH, their contents were determined by an assay kit, and another kit was used to determine the NADP(H) content according to the manufacturer’s instructions (Comin Biotechnology Co., Ltd., Suzhou, China).

### ATP and ADP Measurements

The ATP and ADP contents were determined using ATP and ADP assay kits according to the manufacturer’s instructions (Shanghai Enzyme-linked Biotechnology Co., Ltd., China). During this process, 0.1 g of frozen leaves were homogenized with 1 mL of 0.1 M PH7.4 PBS in an ice bath and centrifuged at 3000×*g* for 20 min. The supernatant was collected for analysis at 450 nm.

### ATPase Content

ATPase content was determined with by the ELISA method and an assay kit according to the manufacturer’s instructions (Shanghai Enzyme-linked Biotechnology Co., Ltd., China). During this process, 0.1 g of frozen leaves were extracted with 0.1 M PH7.4 PBS, and then centrifuged at 3000×*g* for 20 min at 4 °C. The supernatant was collected for analysis at 450 nm.

### PARP Content

PARP content was determined by ELISA method and an assay kit according to the manufacturer’s instructions (Shanghai Enzyme-linked Biotechnology Co., Ltd., China). During this process, 0.2 g of frozen leaves were extracted with 0.1 M PH7.4 PBS, and then centrifuged for 20 min at 3000×*g*. The supernatant was collected for analysis at 450 nm.

### Quantitative Real-Time Polymerase Chain Reaction (PCR) Analysis

Total RNA was extracted from 0.3 g leaves using TRIpure reagent (Aidlab Biotechnologies, Beijing, China). RNA was converted to first-strand cDNA using ReverTra Ace qPCR RT Master Mix (TOYOBO, Shanghai, China). The SYBR Green I (TOYOBO) was used as a fluorescent reporter, and the resultant cDNA was used as a template for quantitative PCR amplification in a Thermal Cycler Dice Real Time System II (TaKaRa Biotechnology, Dalian, China). Primers were designed using PRIMER5 software (Rozen and Skaletsky 2000). The primers for genes examined were listed in Supplementary Table 1. The PCR and detection were performed as described above (Feng et al. 2013). Relative transcript levels were analyzed using 2^−ΔΔCT^method and the experiments were performed in triplicate.

### Statistical Analysis

Data were processed using SPSS software 11.5 (IBM Corp., Armonk, NY, USA) to detect differences. The mean values and standard errors in the figures represented data from three experimental replicates unless otherwise stated. The *t-*test was performed on the normalized data. An analysis of variance (ANOVA) with two factors (temperature and treatment) was used to compare the differences in LSD test with *p* (*p* ≤ 0.05).

## Results

### Changes of Leaf Morphology, Photosynthesis and REC under Cold Stress

**Fig. 1:**
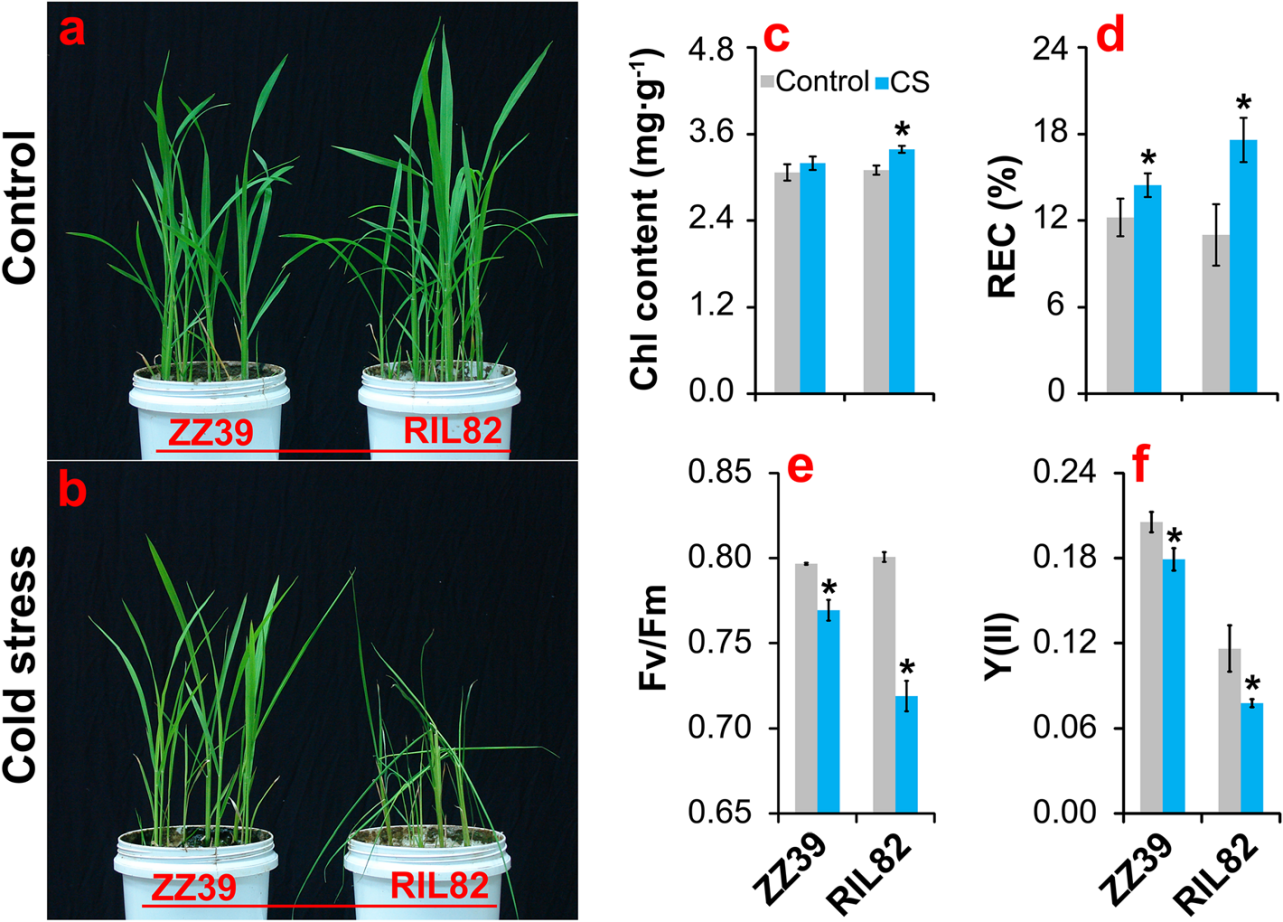
The response of rice plants to cold stress. **a** and **b**, The photograph of ZZ39 and RIL82 plants under control and cold stress conditions, respectively; **c**, Chlorophyll content; **d**, REC; **e**, Fv/Fm; **f**, Y (II). Chl, Chlrophyll; REC, Relative electrical conductance; Fv/Fm, Maximum fluorescence quantum of PSII; Y (II), Actual fluorescence quantum of PSII. Vertical bars denote standard deviations (*n* = 4). A *t*-test was adopted to compare the difference between control and cold stress within a cultivar. * denotes *P* < 0.05

Rice plants ZZ39 and RIL82 showed different responses to cold stress (Fig. 1). Under control conditions, there was no difference in leaf morphology between the two rice plants. However, the leaves of RIL82 plants withered under cold stress, while the leaves of ZZ39 plants remained flat (Fig. 1a, b). The chlorophyll content of the leaves of ZZ39 plants maintained constant under cold stress, but it increased significantly in RIL82 plants compared with control (Fig. 1c). Similarly, higher increase in REC of leaf was found in RIL82 than ZZ39 plants under cold stress (Fig. 1d). In contrast, both Fv/Fm and Y (II) values deceased significantly in response to cold stress, and RIL82 plants decreased more than ZZ39 plants (Fig. 1e, f).

### H_2_O_2_ and MDA Contents

**Fig. 2:**
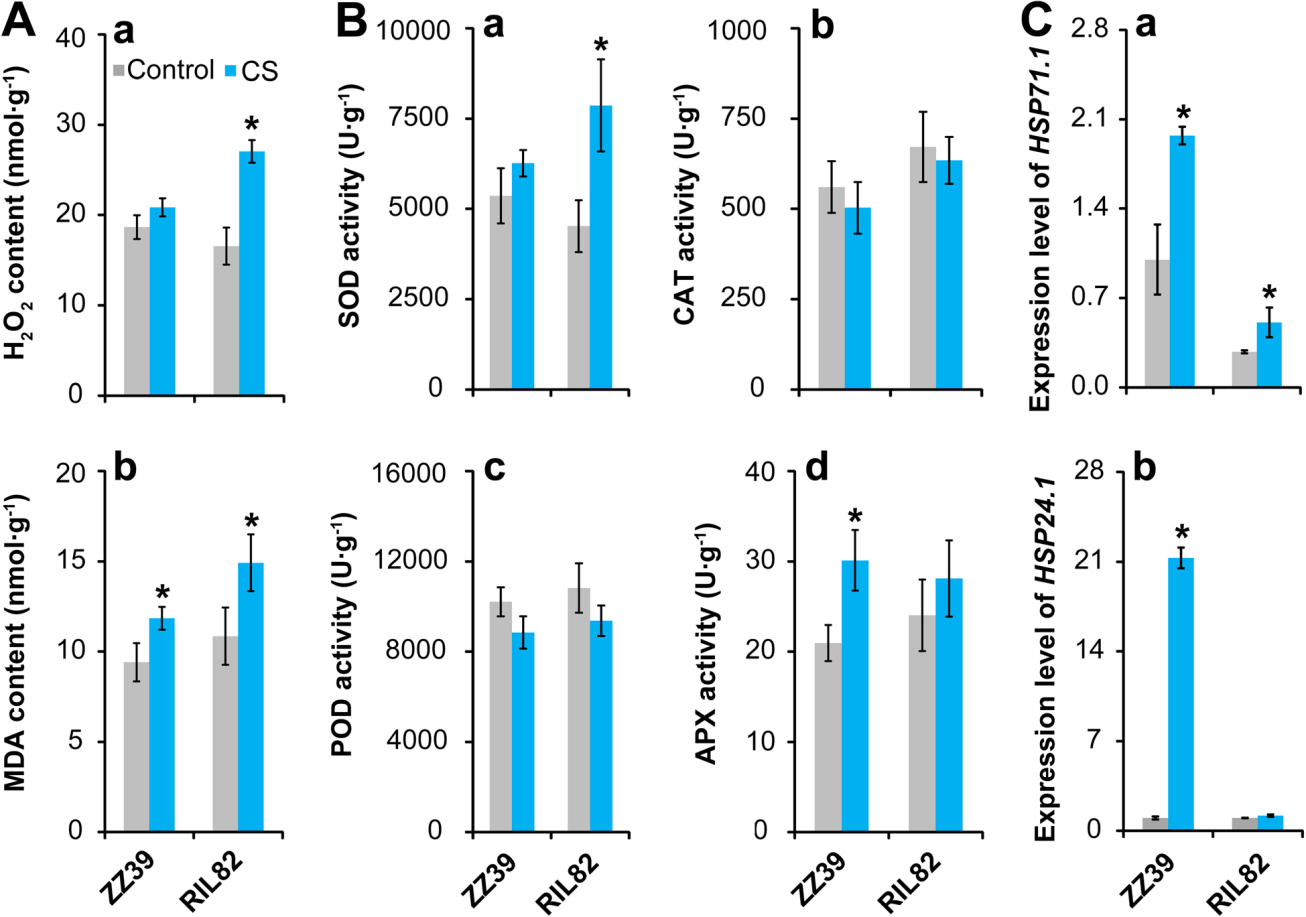
Effects of cold stress on antioxidant enzyme activities, H_2_O_2_ and MDA contents and heat shock proteins in rice leaves. **a**, H_2_O_2_ and MDA; **b**, Antioxidant enzyme activities; **c**, Expression levels of heat shock proteins. Vertical bars denote standard deviations (*n* = 3). A *t*-test was conducted to compare the difference between control and cold stress within a cultivar. * denotes *P* < 0.05

The H_2_O_2_ content in the leaves of ZZ39 plants was not affected by cold stress as there was no significant difference between the control and cold stress groups (Fig. 2A, a). However, the H_2_O_2_ content in RIL82 plants increased significantly in response to cold stress. The MDA content of both plants increased significantly under cold stress (Fig. 2A, b). Compared with the control groups, a greater increase in MDA content was found in RIL82 than ZZ39 plants under cold stress.

### Antioxidant Enzyme Activities

The activities of SOD, POD, CAT and APX were determined to investigate the effects of cold stress on the antioxidant capacity (Fig. 2B). No difference in SOD activity was found between the control and cold stress groups of ZZ39 plants, while a significant increase in SOD activity was observed in RIL82 plants under cold stress (Fig. 2B, a). The activities of POD and CAT were not affected by cold stress as no differences were showed between the control and cold stress groups (Fig. 2B, b and c). However, the APX activity of ZZ39 plants increased significantly under cold stress, while no significant difference was showed between the control and cold stress groups of RIL82 plants (Fig. 2B, d).

### Heat Shock Proteins

The genes associated with heat shock proteins were determined, such as *HSP71.1* and *HSP24.1* (Fig. 2C). The expression level of *HSP71.1* was significantly induced by cold stress in both rice plants, where higher increase was found in ZZ39 than RIL82 plants (Fig. 2C, a). Compared with the control, about 26-fold increase in expression level of *HSP24.1* was showed in ZZ39 plants under cold stress, while no difference was found in RIL82 plants between the control and cold stress groups (Fig. 2C, b).

### GSH Metabolism

**Fig. 3:**
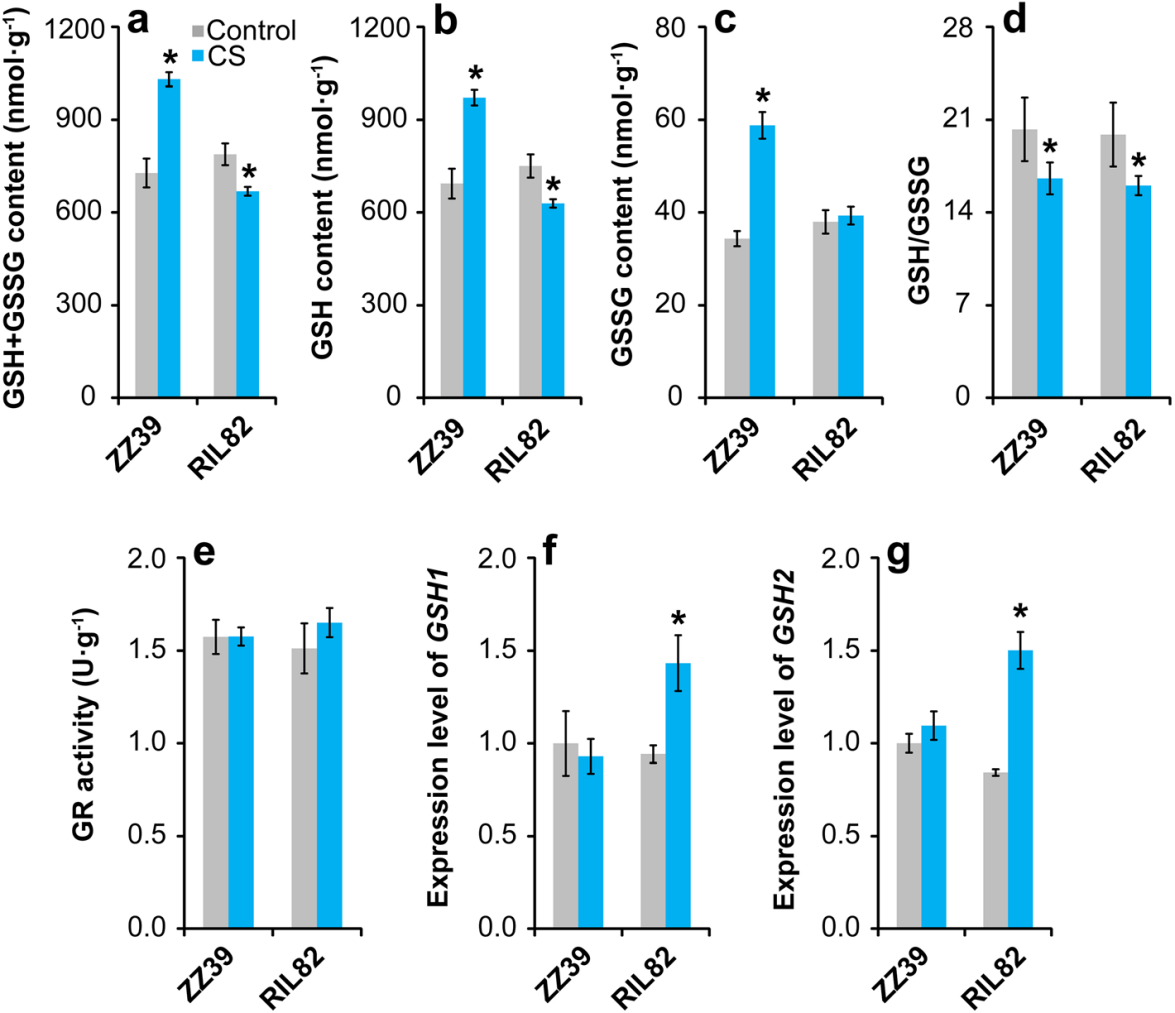
Effect of cold stress on the GSH metabolism in rice leaves. **a**, GSH + GSSG; **b**, GSH; **c**, GSSG; **d**, GSSG/GSH; **e**, GR activity; **f**, *GSH1*; **g**, *GSH2*. GSH, glutathione; GSSG, glutathione disulphide (Oxidant glutathione); GR, glutathione reductase. Vertical bars denote standard deviations (*n* = 4). A t-test was conducted to compare the difference between control and cold stress within a cultivar. * denotes *P* < 0.05

According to the above results, APX was mainly responsible for reducing the H_2_O_2_ and MDA levels caused by cold stress, which was presumably related to GSH. Therefore, the metabolism of GSH was determined under cold stress. Compared with the control, the contents of GSH + GSSG, GSH, and GSSG in the leaves of ZZ39 increased significantly under cold stress, while they decreased clearly in RIL82 plants except for the GSSG (Fig. 3a-c). Regarding the GSH/GSSG, it was significantly reduced by cold stress, but no obvious difference in decrease was showed between these two rice plants (Fig. 3d). Additionally, there was no obvious difference in GR activity between the control and cold stress groups in both rice plants (Fig. 3e). Similarly, no significant differences in the expression levels of *GSH1* and *GSH2* of leaves in ZZ39 plants were showed between the control and cold stress groups (Fig. 3f, g), while they increased significantly in RIL82 plants under cold stress.

### Energy Production and Consumption

**Fig. 4:**
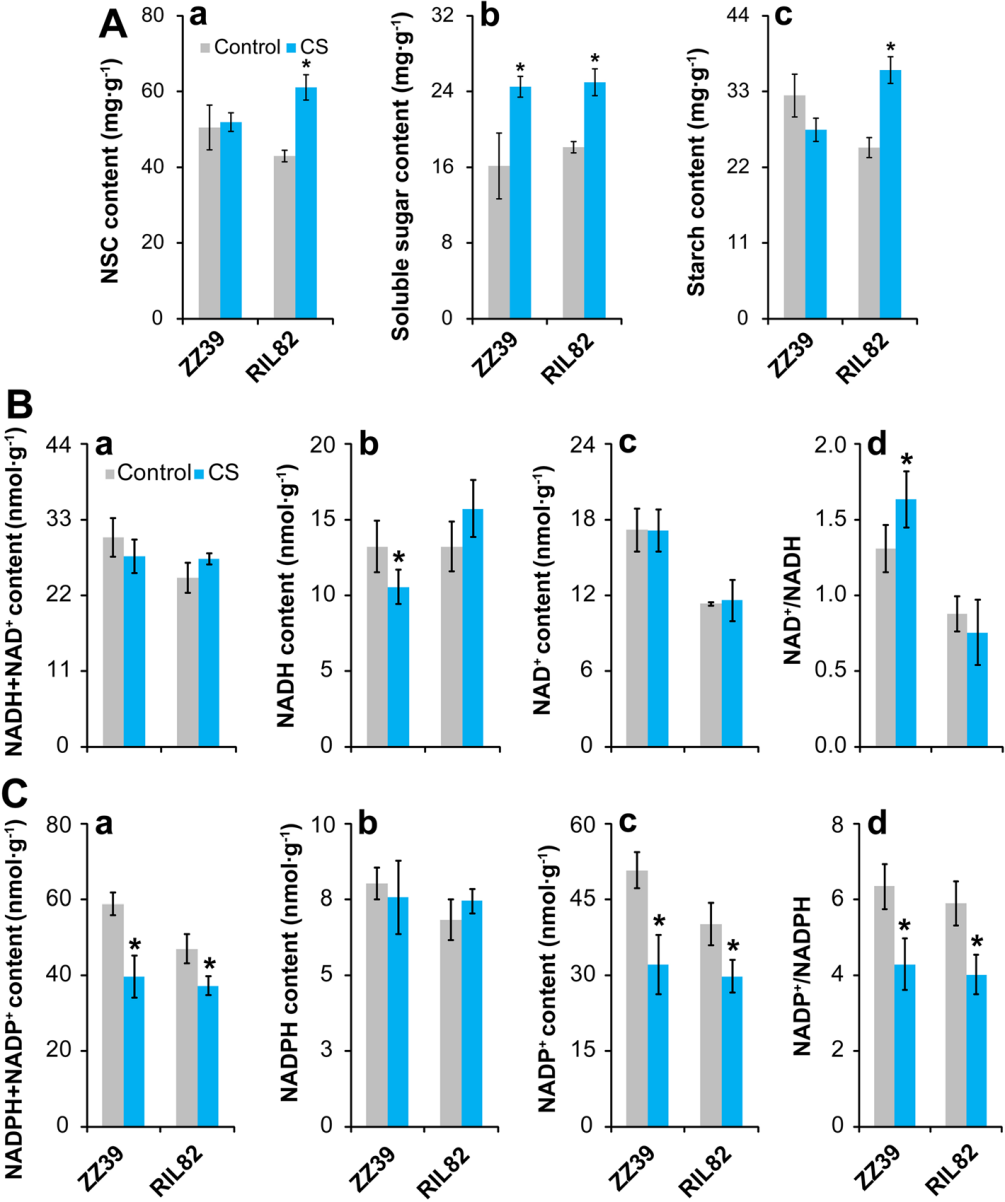
Effects of cold stress on carbohydrate, NAD(H) and NADP(H) contents in rice leaves. **a**, Carbohydrates; **b**, NAD(H) metabolism; **c**, NADP(H) metabolism. NSC, Non-structural carbohydrates; NAD(H), Nicotinamide adenine dinucleotide; NADP(H), Nicotinamide adenine dinucleotide phosphate. Vertical bars denote standard deviations (*n* = 4). A *t*-test was conducted to compare the difference between control and cold stress within a cultivar. * denotes *P* < 0.05

The energy status is important for GSH accumulation in RIL82 plants under cold stress, since the process of GSH synthesis is dependent on ATP. Carbohydrates, including NSC, soluble sugars and starch are fundamental substrates for energy production such as NAD(H), NADP(H) and ATP, thus their contents were determined in plants under cold stress. Cold stress caused a few effect on the NSC content of ZZ39 plants, while a remarkable increase was found in RIL82 plants under cold stress compared with control (Fig. 4A, a). In response to cold stress, the soluble sugar content increased significantly in both rice plants, while higher increase was found in ZZ39 than RIL82 plants compared with control (Fig. 4A, b). Interestingly, the starch content in leaves of ZZ39 decreased under cold stress, while a large increase was showed in RIL82 plants (Fig. 4A, c).

Cold stress caused a few effect on the contents of NAD^+^+NADH and NAD^+^, since there were no obvious differences between the control and cold stress groups in either plants (Fig. 4B, a and c). The NADH content of ZZ39 plants significantly decreased under cold stress, while slight increase was found in RIL82 plants compared with control (Fig. 4B, b). In contrast, the NAD^+^/NADH increased significantly in ZZ39 plants in response to cold stress, while no obvious difference was showed between the control and cold stress group in RIL82 plants (Fig. 4B, d). As to the contents of NADP^+^+NADPH and NADP^+^ as well as NAD^+^/NADPH, they decreased significantly in ZZ39 and RIL82 plants under cold stress, and higher decreases were found in the former than the latter (Fig. 4C, a, c and d). In contrast, the differences in NADPH between the control and cold stress groups were not significant in two rice plants (Fig. 4C, b).

**Fig. 5:**
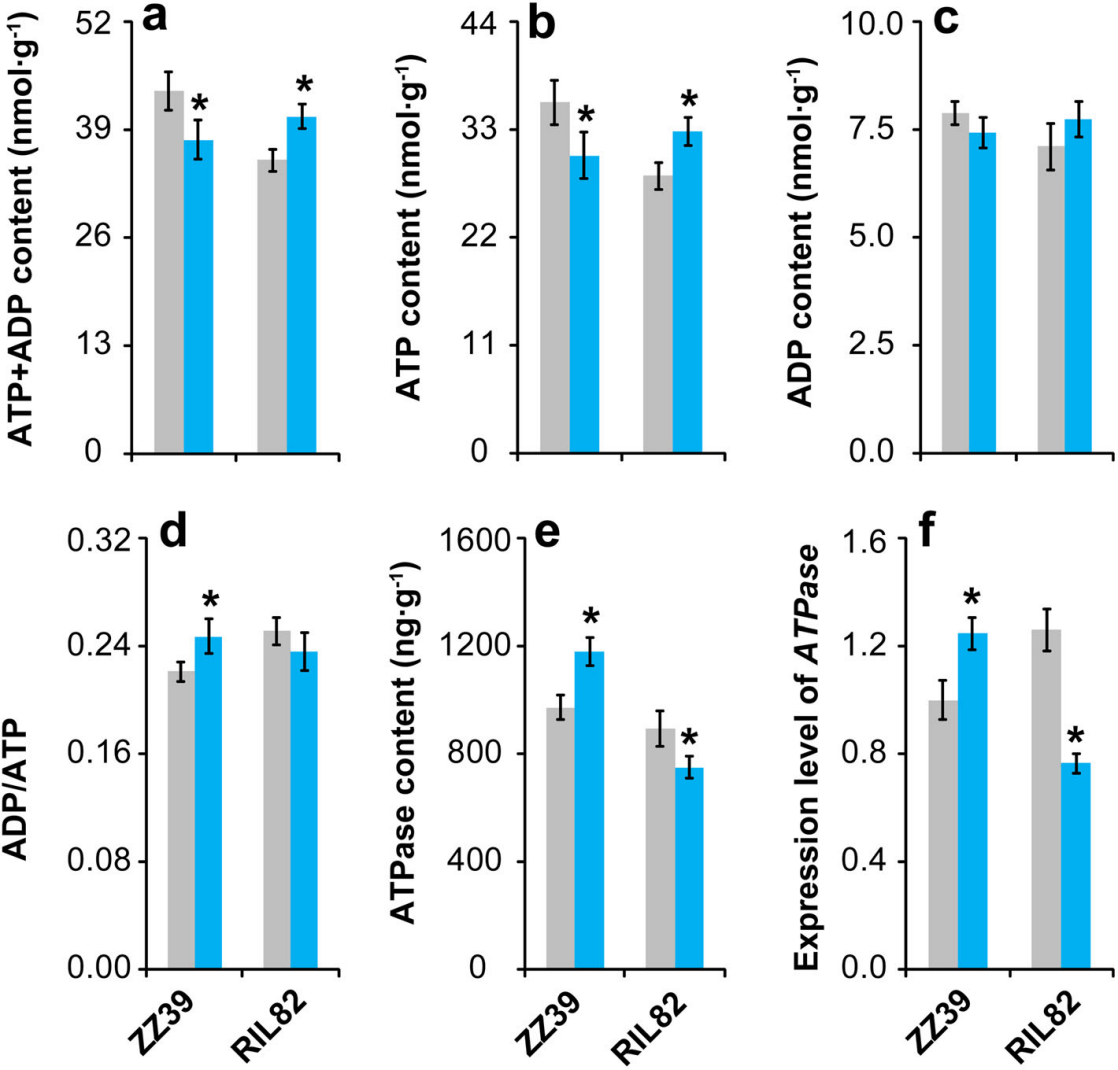
Effect of cold stress on the ATP metabolism in rice leaves. **a**, ATP + ADP; **b**, ATP; **c**, ADP; **d**, ADP/ATP; **e**, ATPase; **f**, expression level of *ATPase*. Vertical bars denote standard deviations (*n* = 3). A *t*-test was conducted to compare the difference between control and cold stress within a cultivar. * denotes *P* < 0.05

Under cold stress, the contents of ATP + ADP, ATP and ADP in ZZ39 plants decreased compared with their respective controls. However, they increased in RIL82 plants under cold stress with significant differences showed in ATP + ADP and ATP (Fig. 5a-c). In contrast, the ADP/ATP, ATPase content and expression level of *ATPase* in ZZ39 plants increased clearly in response to cold stress, while a great reduction was found in RIL82 plants except for ADP/ATP (Fig. 5d-f).

### PARP Content and Expression Levels of Genes Associated with SnRK1 and TOR

**Fig. 6:**
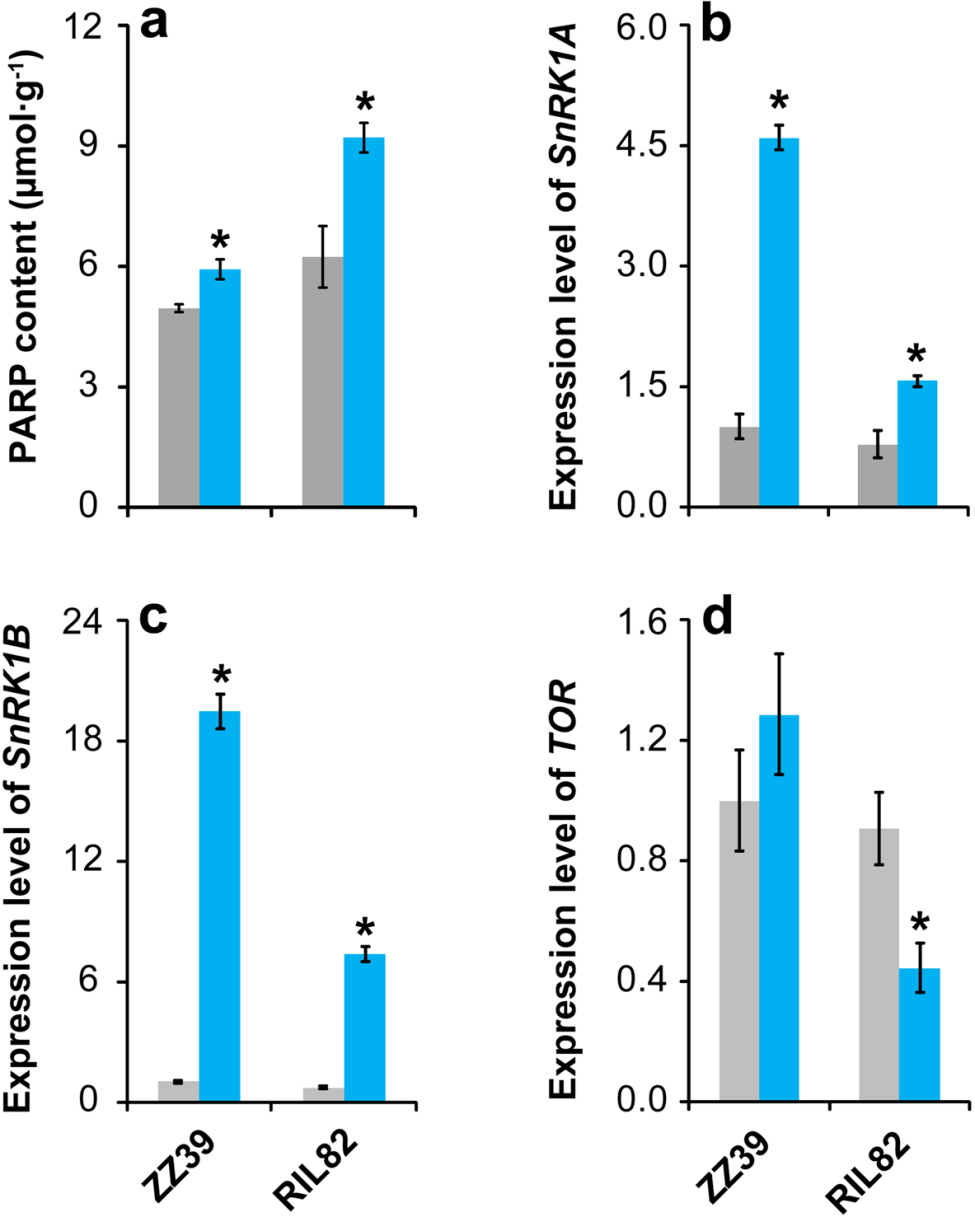
Effects of cold stress on PARP content and expression levels of genes related to *SnRK1* and *TOR* in rice leaves. PARP, Poly (ADP-ribose) polymerase; SnRK1, SNF1-related protein kinase; TOR, Target of rapamycin*.* Vertical bars denote standard deviations (*n* = 3). A *t*-test was conducted to compare the difference between control and cold stress within a cultivar. * denotes *P* < 0.05

The PARP content of both plants significantly increased under cold stress, and higher increase was found in RIL82 than ZZ39 plants compared with control (Fig. 6a). The *SnRK1*A and *SnRK1B* genes were significantly expressed by cold stress, in which the increase in ZZ39 plants was higher than that in RIL82 plants (Fig. 6b, c). Compared with control, the expression level of *TOR* of ZZ39 plants increased slightly under cold stress, while a large decrease was showed in RIL82 plants (Fig. 6d).

### The Role of Energy Status in Plants in GSH Accumulation under Cold Stress

**Fig. 7:**
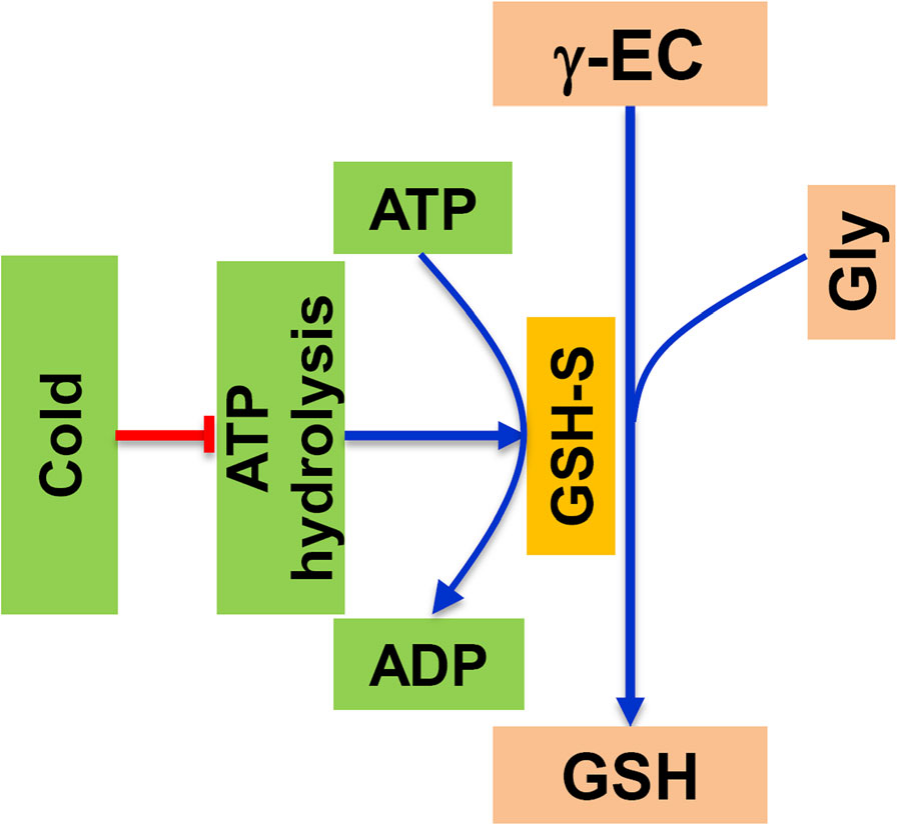
Descriptive model of the mechanism of cold stress reducing GSH by inhibiting ATP hydrolysis in rice leaves. The γ-EC and Gly is converted into GSH by Glutathione synthetase (GSH-S) in plants. This pathway requires energy consumption (ATP). Under cold stress, ATP content significantly increases in the cold susceptible cultivar, indicating that it is not inhibited by stress. In contrast, cold stress inhibits the hydrolysis of ATP, resulting in low GSH accumulation, thereby reducing available energy. γ-EC, γ-glutamylcysteine; Gly, Glycine

According to above results, energy deficit was not the main limiting factor for the lower GSH accumulation in RIL82 plants caused by cold stress, because the ATP content in RIL82 plants increased significantly under cold stress, while it clearly decreased in ZZ39 plants. Under cold stress, the increases in expression levels of *GSH1* and *GSH2* in RIL82 plants were higher than those in ZZ39 plants. However, the increases in ATPase content and *ATPase* expression level of ZZ39 plants were significantly higher than those of RIL82 under cold stress. We inferred that inhibition of ATP hydrolysis by cold stress might be the main constraint factor for GSH accumulation in cold-sensitive genotype RIL82 (Fig. 7 and Fig. S1). Therefore, GSH and 3-ab were sprayed separately or together onto rice plants to confirm the hypothesis that energy utilization ability is related to the accumulation of GSH in plants under cold stress.

### Effect of GSH, BSO or 3-Ab on Rice Plants under Cold Stress

**Fig. 8:**
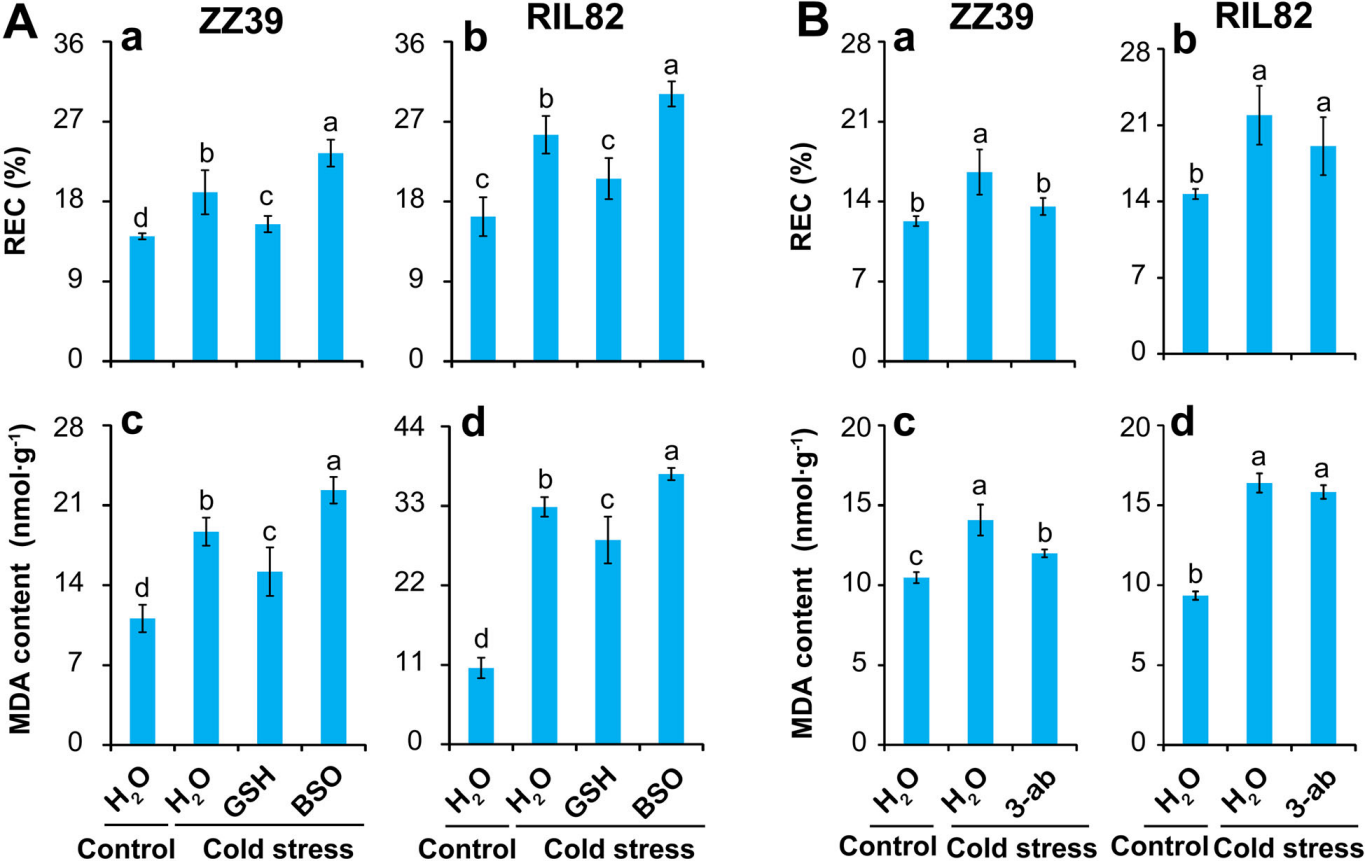
Effect of GSH, BSO, or 3-ab on the contents of REC and MDA in rice leaves under cold stress. GSH, Glutathione; BSO, Buthionine sulfoximine; 3-ab, 3-aminobenzamide; REC, relative electric conductance. Vertical bars denote standard deviations (*n* = 3). Different letters indicate significant differences among the treatments under control and cold stress within a genotype by two-way analysis of variance for two factors (temperature and treatment) (*P* < 0.05)

In response to cold stress, the REC of both genotypes increased significantly (Fig. 8A, a and b). However, GSH reversed this effect, since the REC in leaves of plants treated with GSH was significantly lower than that of H_2_O treatment under cold stress. Accordingly, rice plants treated with BSO had the highest REC among all treatments in both rice plants under cold stress. Similarly, MDA significantly increased in both rice plants under cold stress, among which GSH-treated plants had the lowest content, while BSO treatment had the highest value (Fig. 8A, c and d). Compared with the plants treated with H_2_O, a remarkable reduction in REC and MDA was found in the plants of ZZ39 treated with 3-ab under cold stress (Fig. 8B). However, such results were not found in RIL82 plants, as no significant difference was showed between the treatments of H_2_O and 3-ab under cold stress.

### Effects of GSH and 3-Ab Combination on Rice Plants under Cold Stress

**Fig. 9:**
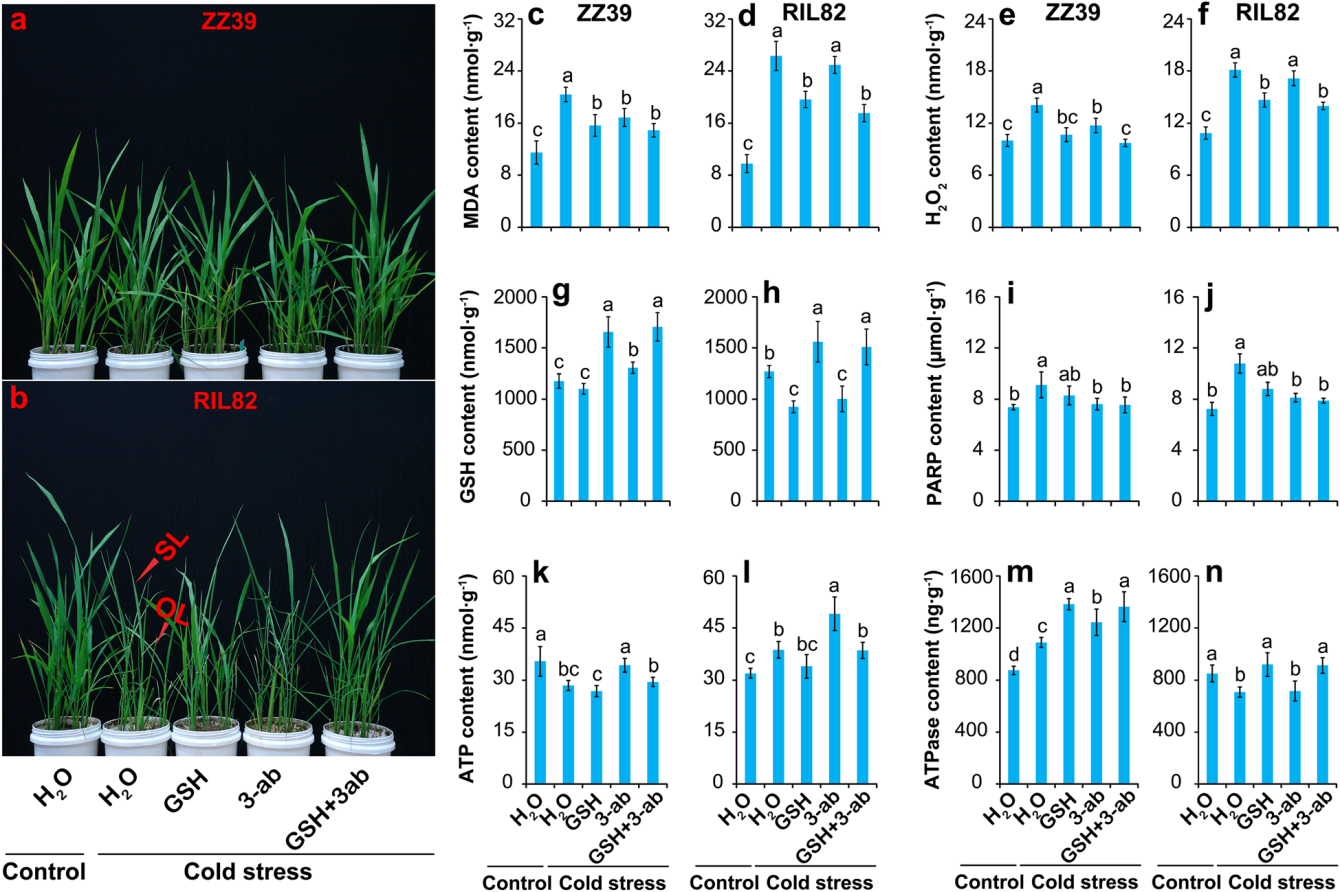
Effects of GSH and 3-ab on leaf morphology and contents of MDA, H_2_O_2_, GSH, ATP, PARP and ATPase in rice leaves under cold stress. **a** and **b** The photos of leaf morphology; **c** and **d**, MDA; **e** and **f**, H_2_O_2_; **g** and **h**, GSH; **i** and **j**, PARP; **k** and **l**, ATP; **m** and **n**, ATPase. GSH, Glutathione; 3-ab, 3-aminobenzamide; GSH, Glutathione; PARP, Poly (ADP-ribose) polymerase; SL, Second leaf; OL, Old leaf. Vertical bars denote standard deviations (*n* = 3). Different letters indicate significant differences among the treatments under control and cold stress within a genotype by two-way analysis of variance for two factors (temperature and treatment) (*P* < 0.05)

The above results indicated that exogenous GSH enhanced cold tolerance in these two rice plants, while such result was only found in ZZ39 plants when treated with PARP inhibitor (3-ab). Thus, we wonder whether there is a synergistic effect between GSH and 3-ab in enhancing cold tolerance in plants. According to the photos, the leaves of ZZ39 treated with H_2_O or 3-ab wilted slightly under cold stress, while the plants treated with GSH or GSH + 3-ab maintained flat (Fig. 9a, b). In contrast, the leaves of RIL82 plants treated with H_2_O and 3-ab severely wilted under cold stress, whereas these effects were reversed by GSH or GSH + 3-ab, especially for the old leaves (Fig. S2).

Under cold stress, similar changing patterns of MDA and H_2_O_2_ were found in plants treated with GSH or 3-ab alone (Fig. 9c-f). Additionally, the lowest MDA and H_2_O_2_ levels were showed in the plants treated with GSH + 3-ab under cold stress. Compared with 3-ab treatment, slight decreases in MDA and H_2_O_2_ levels were showed in ZZ39 plants treated with GSH + 3-ab, while a remarkable reduction was observed in RIL82 plants. Regarding the GSH content, it clearly increased in plants treated with GSH or GSH + 3-ab treatments compared with H_2_O treatment in both rice plants in response to cold stress (Fig. 9g, h). Indeed, higher GSH content was found in both plants treated with 3-ab than those plants treated with H_2_O, but significant difference was only found in ZZ39 plants.

Rice plants treated with GSH, 3-ab or GSH + 3-ab attained lower PARP content than H_2_O treatment in both rice plants under cold stress (Fig. 9i, j). However, significant difference was only found in the treatments of 3-ab or GSH + 3-ab compared with H_2_O under cold stress. As to the ATP, the highest levels were showed in the plants treated with 3-ab in both plants under cold stress, which was significantly higher than other treatments (Fig. 9k, l). Interestingly, the lowest value was observed in GSH treatment, but the difference was not significant compared with H_2_O treatment. Under cold stress, the highest content of ATPase were showed in the treatments of GSH and GSH + 3ab in both rice plants, while the lowest content were found in plants treated with H_2_O and 3-ab (Fig. 9m, n). In ZZ39 plants, lower ATPase content was found in H_2_O treatment than 3-ab treatment under cold stress, while no significant differences between these two treatments were showed in RIL82 plants.

## Discussion

### The Function of GSH in Conferring Cold Tolerance in Rice Plants

The present results indicated that cold stress caused more damages to RIL82 than ZZ39 plants (Fig. 1), since excess MDA and H_2_O_2_ were showed in the former than latter (Fig. 2A). As well known, antioxidant enzymes including SOD, POD, CAT and APX can be induced by abiotic stress to alleviate the oxidant stress in plants (Ahmad et al. 2010; Fu et al. 2016; Zhang et al. 2016). Interestingly, APX was the only antioxidant responsible for maintaining ROS homeostasis in rice plants under cold stress (Fig. 2B). This finding was inconsistent with the results that these antioxidants always worked together to resist oxidant stress (Zhang et al. 2017; Gupta et al. 2018; Naeem et al. 2018; Islam et al. 2019). However, similar results were also presented in the research of Liu et al. (2018), who reported that 5-aminolevulinic acid conferred cold tolerance in plants via enhancing the APX, rather than the CAT, with a large increase in accumulation of GSH and ASA. In this study, remarkable increases were found in contents of GSH + GSSG, GSH and GSSG of ZZ39 compared with RIL82 under cold stress, while such effects were not found in GSH/GSSG and GR (Fig. 3). This suggested that GSH might be the main factor resulting in different cold tolerance between these two rice plants. Importantly, exogenous GSH significantly enhanced cold tolerance in both rice plants, whereas this was impaired by its synthetic inhibitor BSO (Fig. 8A).

### The Role of ATP Hydrolysis in GSH Synthesis in Rice Plants under Cold Stress

The GSH accumulation is determined by glutathione synthetase (GSH-S) and GR in plants, and the former are responsible for the GSH synthesis using the γ-EC and Gly while the latter reduces the GSSG to GSH (Rao and Reddy 2008; Noctor et al. 2012). According to the present results, the GSH-S rather than GR is responsible for GSH accumulation in plants under cold stress (Fig. 3). The genes of *GSH1* and *GSH2* encoding γ-ECS and GSH-S respectively are mainly responsible for the synthesis of GSH in plants (Cairns et al. 2006; Pasternak et al. 2008). However, higher increases in expression levels of *GSH1* and *GSH2* were showed in RIL82 than ZZ39 plants under cold stress (Fig. 3). These paradoxical results might be mainly ascribed to the energy status in plants under cold stress, since these two pathways is ATP dependent (Buwalda et al. 1990; Noctor et al. 1997; Ogawa et al. 2004).

Energy homeostasis is important for plants to survive in abiotic stress, which always result in energy shortage to inhibit the plants growth and development (De Block and Van Lijsebettens 2011; Xiong and Sheen 2015; Zhang et al. 2018b; Islam et al. 2019; Rodriguez et al. 2019). However, it was the energy ultilization ability rather than energy shortage that mainly contributed to the different cold tolerance between these two rice plants (Figs. 4 and 5). The ATPase content and its expression level increased significantly under cold stress in ZZ39 plants, while a large decrease was found in RIL82 plants (Fig. 4). This suggested that ATP hydrolysis in RIL82 plants were adversely inhibited by cold stress, and thus the lower GSH synthesis because of the higher unavailable ATP (Puhakainen et al. 1999; Mendoza et al. 2000; Deng et al. 2015; Muzi et al. 2016). This hypothesis was confirmed by the present results that the PARP inhibitor (3-ab) only enhanced the cold tolerance and GSH content in ZZ39 plants under cold stress (Fig. 8B and 9), though the ATP content increased significantly in both rice plants (Fig. 9k and l).

It is puzzling that the ATP hydrolysis increased in RIL82 plants under cold stress in the present of exogenous GSH (Fig. 9n). This indicated that exogenous GSH could activate ATPase to provide energy for the GSH synthesis under cold stress. Similar results have not been documented previously that how GSH activating ATPase in plants under cold stress remains unclear.

### The Energy Allocation for Rice Plants to Survive in Cold Stress

It has been reported that the ATP synthetic rates are adversely inhibited in abiotic stress conditions (Gibbs and Greenway 2003), where higher rates of glycolysis and activities of fermentative enzymes were observed in plants (Gibbs et al. 2000; Saika et al. 2006). In this case, the complementary responses could be used by the plants with low energy status to stabilize energy charge, including that ATP-regenerating pathways such as glycolysis become derepressed to maximize energy production and retard ATP-utilizing pathways to conserve ATP (Gibbs et al. 2000). In this study, more energy consumption was found in the ZZ39 plants than RIL82 under cold stress (Figs. 4 and 5). This strategy was not beneficial for plants to resist cold stress. However, there is a hierarchical down-regulation of ATP consumption during periods of ATP shortage (Atwell et al. 1982; Gibbs and Greenway 2003), in which the protein consumed the largest proportion of ATP synthesis (Edwards et al. 2012). This explained the remarkable decrease in ATP in ZZ39 plants, but higher increases in content of GSH and expression levels of heat shock proteins than RIL82 plants under cold stress. Thus, we inferred that the ZZ39 plants consumed more energy for the synthesis of GSH and heat shock proteins to resist cold stress, rather than the plant growth and development.

Heat shock protein is always accumulated to confer heat tolerance in plants under heat stress (Merret et al. 2017; Islam et al. 2019; Jiang et al. 2020; Li et al. 2020). It is worth noting that, similar results were also found in the rice plants under cold stress, in which higher increase in expression levels of *HSP71.1* and *HSP24.1* were found in the cold resistant plants than the cold susceptible one compared with their respective controls (Fig. 2C). This finding is consistent with the previous results that the accumulation of heat shock proteins is also found in plants under drought, salt, and oxidant stress (Kim et al. 2012; Jacob et al. 2017; Zhang et al. 2018c; Zandalinas et al. 2018). Actually, in addition to its protective effect under stress conditions, the heat shock protein also plays a role in plant development under normal growth conditions (Neta-Sharir et al. 2005).

SnRK1 and TOR which act in opposite ways in the regulation of metabolic-driven processes, play central roles in balancing energy requirements with supplies for plants to survive in unfavorable conditions (Baena-González and Hanson 2017; Crepin and Rolland 2019; Margalha et al. 2019). Without exception, notably higher increases in expression levels of *SnRK1A* and *SnRK1B* were found in ZZ39 than RIL82 plants under cold stress (Fig. 6b, c), which was consistent with the previous results (Valledor et al. 2013; Lin et al. 2014; Yu et al. 2018). However, a large decrease in expression level of *TOR* was showed in RIL82, rather than ZZ39 plants under cold stress (Fig. 6d). Clearly, this changing pattern between *SnRK1* and *TOR* don’t follow the “yin-Yang” model (Rodriguez et al. 2019). It has been reported that the TOR can be activated to induce the synthesis of GSH and heat shock proteins and confer cold and drought tolerance in plants (Dobrenel et al. 2013; Xiong and Sheen 2015; Bakshi et al. 2017; Speiser et al. 2018; Rodriguez et al. 2019). This suggests that the antagonism between the *SnRK1* and *TOR* may be ambiguous and the kinases may act in a different way under certain physiological circumstances (Rodriguez et al. 2019). The target genes of TOR and SnRK1 kinases only partially and not always antagonistically overlay under energy deficiency (Wu et al. 2019). Additionally, the TOR was reported to be activated by ATPase (Zoncu et al. 2011), which could explain the lower expression level of *TOR* showed in RIL82 than ZZ39 under cold stress. Therefore, we inferred that the ATPase might function in the process of SnRK1 and TOR acting together to regulate the energy homeostasis in plants under cold stress.

## Conclusion

Cold stress caused more damages to RIL82 than ZZ39 plants, since higher increases in REC, MDA and H_2_O_2_ were found in the former than the latter. Among the antioxidants including SOD, POD, CAT, APX, GSH, and GR, there were only APX and GSH involved in regulating cold tolerance between the two rice plants. The APX activity and GSH content increased significantly in ZZ39 plants under cold stress, while in RIL82 plants no obvious differences were showed between the control and cold stress. However, significantly higher increases in expression levels of *GSH1* and *GSH2* as well as contents of carbohydrates, NAD(H), NADP(H) and ATP were found in RIL82 under cold stress, rather than the ZZ39 plants. These findings indicated that lower GSH accumulation in RIL82 plants was not due to the energy deficit caused by cold stress. It’s worth noting that, the ATPase content and its expression level increased obviously in ZZ39 plants under cold stress, while a remarkable decrease was found in RIL82 plants. This suggested that the ATP hydrolysis by ATPase play a key role in GSH accumulation. Therefore, we inferred that the ATPase was the main factor responsible for determining cold tolerance between these two rice plants via regulating the GSH accumulation.

## Supplementary information

**Additional file 1: Table S1.** Primer sequences used in quantitative Real-Time reverse transcription PCR. **Figure S1.** Descriptive model of relationships among the GSH accumulation, heat shock protein and energy homeostasis in plants under cold stress. The GSH plays a key role in reducing ROS by regulating the APX activity in plants, which can alleviate cold damage. During this process, the accumulation of GSH is determined by GSH-S and GR, the former consumes ATP, while the latter consumes NADPH. The heat shock protein can be induced by ROS, which in turn reduce excess ROS in plants. Indeed, the accumulation of heat shock protein is a process of high energy consumption via consuming ATP. Thus, PARP which can be activated by ROS could inhibit the accumulation of heat shock proteins because it can consume NAD^+^ and thereby reduce ATP under cold stress. γ-EC, γ-glutamylcysteine; Gly, Glycine; GSH, Glutathione; 3-ab, 3-aminobenzamide; GSH, Glutathione; PARP, Poly (ADP-ribose) polymerase. **Figure S2.** The morphology of the second and old leaves in RIL82 plants under cold stress. SL, Second leaf; OL, Old leaf.

## Data Availability

The datasets supporting the conclusions of this article are included in the article (and its additional files).

## Abbreviations

3-ab: 3-aminobenzamide
APX: Ascorbate peroxidase
BSO: Buthionine sulfoximine
CAT: Catalase activity
DHAR: Dehydroascorbate reductase
Fv/Fm: Maximum fluorescence quantum efficiency
CS: Cold stress
GR: Glutathione reductase
GSH: Glutathione
GSH-S: Glutathione synthetase
HSP: Heat shock protein
MDA: Malondialdehyde
MDHAR: Monodehydroascorbic acid reductase
NSC: Non-structural carbohydrates
PARP: Poly (ADP-Ribose) Polymerases
POD: Peroxidase
REC: Relative electrical conductance
ROS: Reactive oxygen species
SOD: Superoxide dismutase
Y (II): Actual fluorescence quantum efficiency
γ-ECS: γ-glutamylcysteine synthetase
