# Supplementary material for: ATP Hydrolysis Determines Cold Tolerance by Regulating Available Energy for Glutathione Synthesis in Rice Seedling Plants
Source: Rice (N Y). 2020 Apr 9;13:23. doi: 10.1186/s12284-020-00383-7 (PMC7145886; doi:10.1186/s12284-020-00383-7)
Supplement: Supplementary file 1 — Additional file 1: Table S1. Primer sequences used in quantitative Real-Time reverse transcription PCR. Figure S1. Descriptive model of relationships among the GSH accumulation, heat shock protein and energy homeostasis in plants under cold stress. The GSH plays a key role in reducing ROS by regulating the APX activity in plants, which can alleviate cold damage. During this process, the accumulation of GSH is determined by GSH-S and GR, the former consumes ATP, while the latter consumes NADPH. The heat shock protein can be induced by ROS, which in turn reduce excess ROS in plants. Indeed, the accumulation of heat shock protein is a process of high energy consumption via consuming ATP. Thus, PARP which can be activated by ROS could inhibit the accumulation of heat shock proteins because it can consume NAD+ and thereby reduce ATP under cold stress. γ-EC, γ-glutamylcysteine; Gly, Glycine; GSH, Glutathione; 3-ab, 3-aminobenzamide; GSH, Glutathione; PARP, Poly (ADP-ribose) polymerase. Figure S2. The morphology of the second and old leaves in RIL82 plants under cold stress. SL, Second leaf; OL, Old leaf. [file 12284_2020_383_MOESM1_ESM.docx]

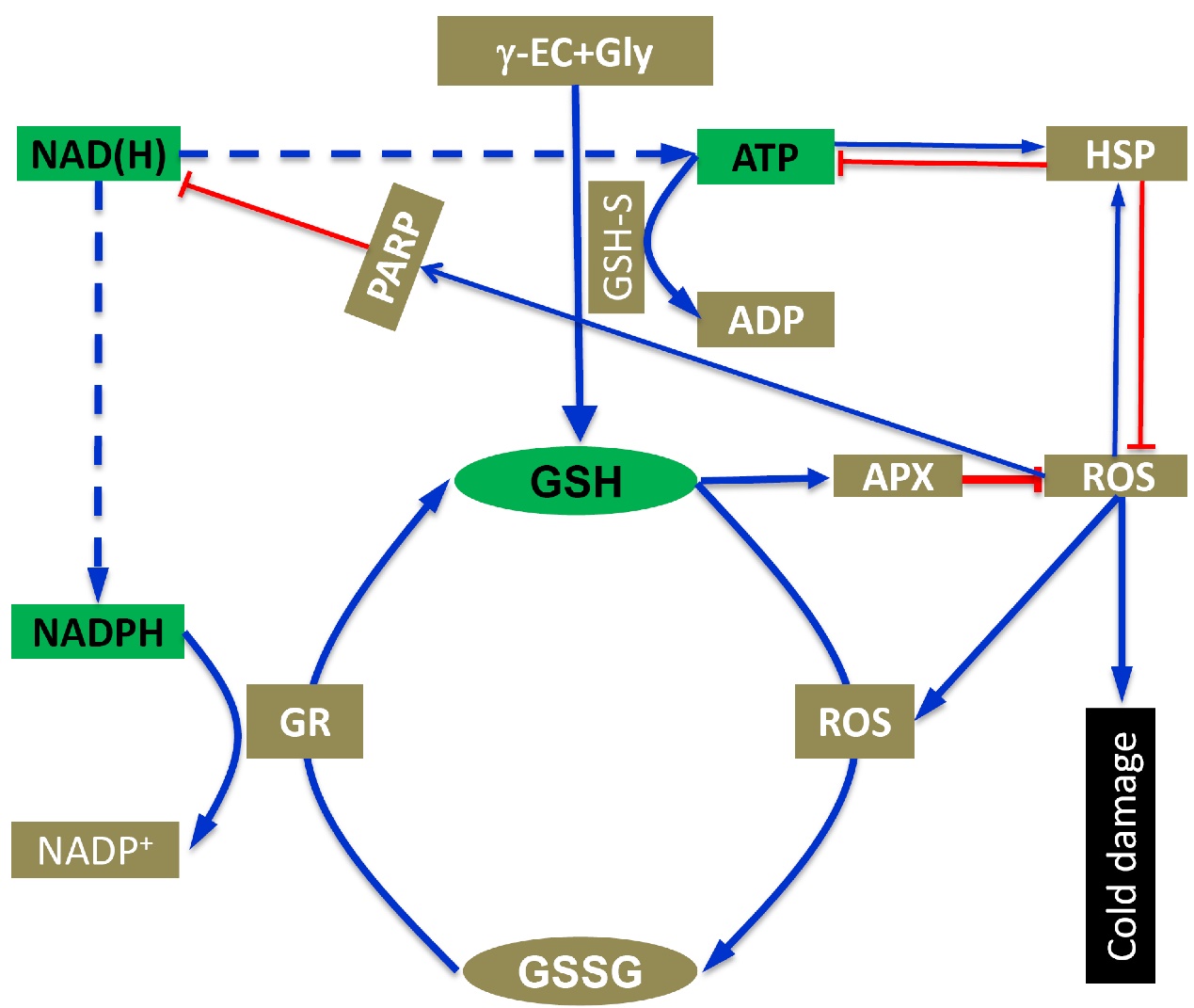


**Fig.S1** Descriptive model of relationships between GSH accumulation, heat shock protein and energy homeostasis in plants under cold stress. The GSH plays a key role in reducing ROS by regulating the APX activity in plants, which could alleviate cold damage. During this process, the accumulation of GSH is determined by GSH-S and GR, the former consumes ATP, while the latter consumes NADPH. The heat shock protein could be induced by ROS, which in turn could reduce excess ROS in plants. Indeed, the accumulation of heat shock protein is a process of high energy consumption via consuming ATP. Thus, PARP can be activated by ROS which could inhibit the accumulation of heat shock proteins because it can consume NAD^+^ and thereby reduce ATP under cold stress. γ-EC, γ-glutamylcysteine; Gly, Glycine; GSH, Glutathione; 3-ab, 3-aminobenzamide; GSH, Glutathione; PARP, Poly(ADP-ribose) polymerase.


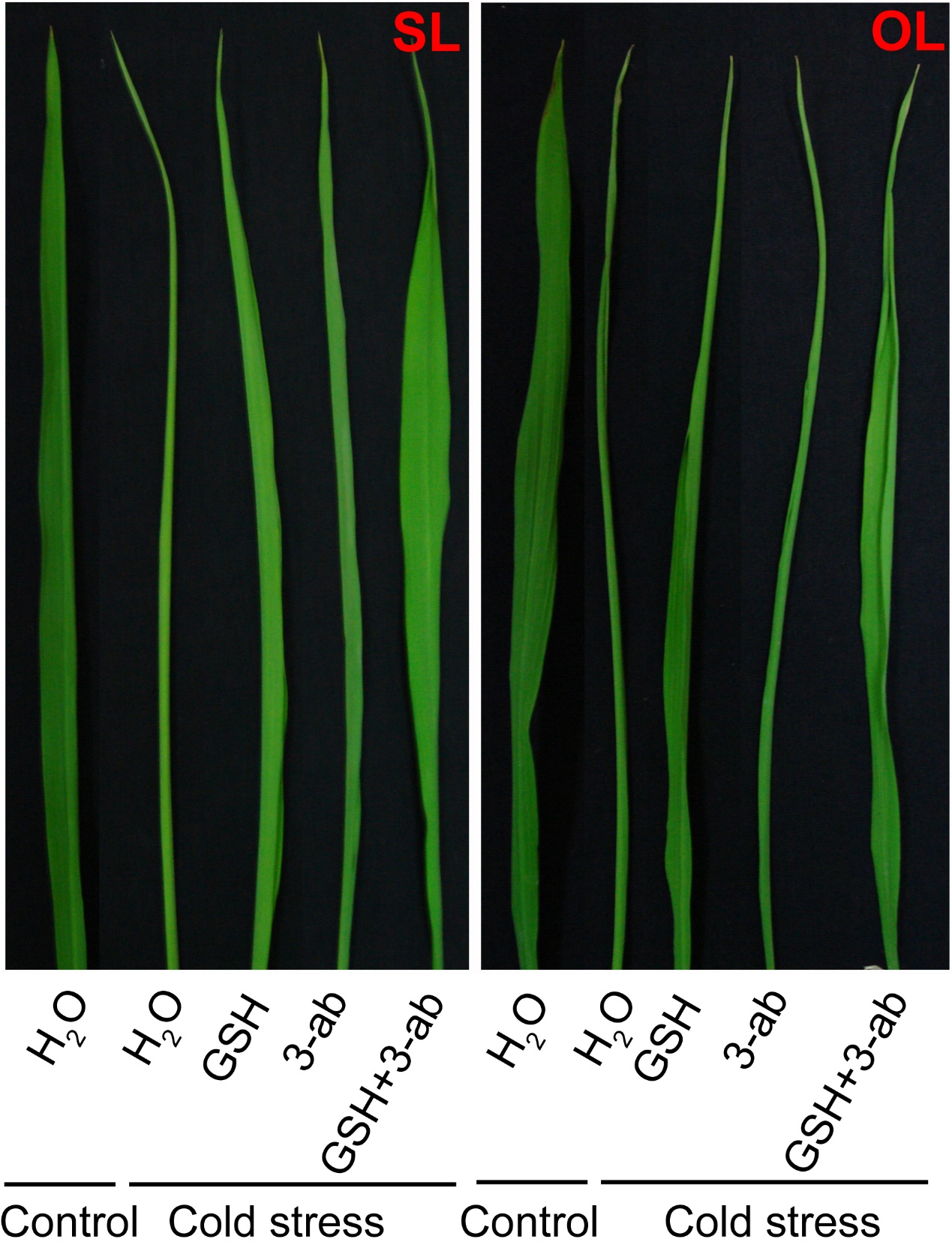


**Fig. S2** The morphology of the second and old leaves in RIL82 plants under cold stress. SL, Second leaf; OL, Old leaf.

**Table S1.** Primer sequences used in quantitative Real-Time reverse transcription PCR.

| Locus ID, gene name | Forward (5’-3’) | Reverse (5’-3’) |
| --- | --- | --- |
| LOC_Os05g45420, *SnRK1A* | ACAACCAGTGGCTACCTTGG | CGATGATCAGTGGCTGAGTT |
| LOC_Os07g09610, *SnRK1B* | ATATCAGGCGCCGAATACTG | TGTGCCTGAAGAACTTGCTG |
| LOC_Os05g14550, *TOR* | GCTGAACGCTGCAATGACTA | ACCGAACAAGTACTGGAGCA |
| LOC_Os03g16860, *HSP71.1* | CTACGAGGGCATCGACTTCT | CGGTGCTCTTGTCCATCTTG |
| LOC_Os02g52150, *HSP24.1* | TGAGCCTCATGGACGACCT | CCCTTGATCACGAGGCTGTT |
| LOC_Os07g27790, *GSH1* | TTCCTGAGGTCAGGCTGAAG | TGGCAAAGCACACAATCTCC |
| LOC_Os05g03820, *GSH2* | GGAGATTGTGTGCTTTGCCA | GGAAGGGTGTCTTCAAACCG |
| NC_001320.1, *ATPase* | TCGGTGGAGCTACTCTTGGA | CGGGCGCGGATCTATGAATA |
| LOC_Os01g22490 ,*UBQ* | GACTACAACATCCAGAAGGAGTC | TCATCTAATAACCAGTTCGATTTC |
